# Evaluation of a Cannabis Harm Reduction Intervention for People With First-Episode Psychosis: Protocol for a Pilot Multicentric Randomized Trial

**DOI:** 10.2196/53094

**Published:** 2023-12-18

**Authors:** Stephanie Coronado-Montoya, Amal Abdel-Baki, José Côté, David Crockford, Simon Dubreucq, Benedikt Fischer, Pamela Lachance-Touchette, Tania Lecomte, Sophie L'Heureux, Clairélaine Ouellet-Plamondon, Marc-André Roy, Ovidiu Tatar, Phillip Tibbo, Marie Villeneuve, Anne Wittevrongel, Didier Jutras-Aswad

**Affiliations:** 1 Department of Psychiatry and Addiction Université de Montréal Montreal, QC Canada; 2 Research Centre Centre Hospitalier de l’Université de Montréal Montréal, QC Canada; 3 Department of Psychiatry Centre Hospitalier de l'Université de Montréal Montreal, QC Canada; 4 Faculty of Nursing Université de Montréal Montreal, QC Canada; 5 Cumming School of Medicine University of Calgary Calgary, AB Canada; 6 Hotchkiss Brain Institute University of Calgary Calgary, AB Canada; 7 Centre for Applied Research in Addiction and Mental Health Simon Fraser University Vancouver, BC Canada; 8 Research & Graduate Studies Division University of the Fraser Valley Abbotsford, BC Canada; 9 Department of Psychiatry Federal University of Sao Paulo Sao Paulo Brazil; 10 School of Population Health University of Auckland Auckland New Zealand; 11 Department of Psychiatry University of Toronto Toronto, ON Canada; 12 Department of Psychology Université de Montréal Montréal, QC Canada; 13 Centre de Recherche de l'Institut Universitaire en Santé Mentale de Montréal Montréal, QC Canada; 14 Clinique Notre-Dame des Victoires Institut Universitaire en Santé Mentale Centre Intégré Universitaire de Soins et Services Sociaux de la Capitale Nationale Québec, QC Canada; 15 Department of Psychiatry and Neurosciences Laval University Québec, QC Canada; 16 Centre de Recherche de l'Institut Universitaire en Santé Mentale de Québec Québec, QC Canada; 17 Centre de Recherche CERVO Québec, QC Canada; 18 Lady Davis Institute for Medical Research Jewish General Hospital Montréal, QC Canada; 19 Department of Psychiatry Dalhousie University Halifax, NS Canada; 20 University Institute on Addictions Montreal, QC Canada

**Keywords:** cannabis, psychosis, harm reduction, pilot, mobile health, psychological intervention, mHealth, young adult, schizophrenia, motivational interviewing, intervention, RCT, randomized, controlled trial, controlled trials, multi-centric, young people, clinical trials, feasibility, perspectives, perspective, evidence-based

## Abstract

**Background:**

Cannabis use is highly prevalent in young people with first-episode psychosis (FEP). Most report cannabis use and are often diagnosed with a cannabis use disorder upon admission to specialized services for psychosis. Cannabis use in this population is associated with worse clinical and psychosocial outcomes, rendering it an important clinical target. Despite this, few cannabis-specific interventions have been developed for FEP and empirically evaluated through randomized controlled trials. Most evaluated interventions have targeted cannabis abstinence, with limited efficacy, but none have centered on harm reduction outcomes for people with FEP who use cannabis. Early intervention services (EIS), the standard of care for FEP, have not successfully addressed problematic cannabis use in people with FEP either. Clinical trials are needed to explore the potential of harm reduction strategies, although these should be preceded by robust pilot studies to establish optimal design and approaches.

**Objective:**

Recognizing the need for harm reduction strategies for individuals with FEP who use cannabis and based on research on patients’ preferences supporting harm reduction interventions, we developed a mobile app–based cannabis harm reduction intervention for this population. This intervention is called Cannabis Harm–reducing Application to Manage Practices Safely (CHAMPS). Here, we describe the protocol for a multicenter, 2-arm, parallel group, randomized pilot trial evaluating the acceptability of CHAMPS for people with FEP who use cannabis and the feasibility of conducting a full-scale trial in this population using CHAMPS. The impact on key clinical outcomes will also be explored.

**Methods:**

This pilot trial aims to recruit 100 young people with FEP using cannabis from 6 Canadian EIS clinics. Participants will be randomized in a 1:1 ratio to CHAMPS+EIS or EIS-only. CHAMPS acceptability will be assessed using completion rates for the intervention arm. Trial feasibility will be assessed using a retention rate for randomized participants. Secondary outcomes will explore tendencies of change in the use of protective behavioral strategies and in motivation to change strategies. Exploratory outcomes include cannabis use–related problems, other substance use, the severity of dependence, psychotic symptoms, and health care service use.

**Results:**

Recruitment began in December 2021. Data collection and analysis are expected to be completed in early 2024. Study results describing CHAMPS acceptability and trial feasibility will then be submitted for publication in a peer-reviewed journal.

**Conclusions:**

CHAMPS uniquely combines evidence-based approaches, patient perspectives, and mobile health technology to support harm reduction in people with FEP who use cannabis. Attaining adequate acceptability and feasibility through this trial may justify further exploration of harm reduction tools, particularly within the context of conducting a larger-scale randomized controlled trial. This pilot trial has the potential to advance knowledge for researchers and clinicians regarding a feasible and user-acceptable research design in the cannabis and early psychosis fields.

**Trial Registration:**

ClinicalTrials.gov NCT04968275, https://clinicaltrials.gov/ct2/show/NCT04968275

**International Registered Report Identifier (IRRID):**

DERR1-10.2196/53094

## Introduction

### Background

Cannabis use is widespread in young people with psychosis, with 60% of young people with first-episode psychosis (FEP) reporting cannabis use at admission into specialized mental health services such as early intervention services (EIS) [1]. Compared to individuals with FEP not using cannabis, people with co-occurring cannabis use are susceptible to worse short- and long-term clinical and mental health outcomes, including persistent psychotic symptoms, more frequent relapses and hospitalizations, and worse psychosocial functioning [1-7]. Given the increased risk of worsened prognosis, it is necessary to deploy evidence-based interventions that target problematic cannabis use (including cannabis use disorder [CUD]) in people with FEP. However, relatively few cannabis-specific interventions have been developed for and tested in people having FEP and those studied have mainly focused on achieving abstinence, showing overall limited efficacy [8-10]. A minority of individuals will recover from CUD without any cannabis-specific treatments [11], and only about half of people with FEP will recover from CUD within 2 years of regular EIS treatment [1]. For many individuals who continue to use cannabis, including those who do not wish to participate in cannabis cessation interventions, more research is needed to determine effective therapeutic options that can reduce potential cannabis use–related harms.

In response to this challenge, there is a growing interest in innovative harm reduction interventions to help individuals with FEP and persistent cannabis use [12-14]. Cannabis harm reduction is an approach that has been endorsed in public health or government reports, as well as by clinicians and young people using cannabis [15-19]. Harm reduction in a drug use context shifts the focus away from the all-or-nothing goal of abstinence and instead focuses on improving the health or quality of life of individuals by targeting the reduction of harmful drug–related consequences [20]. Harm reduction approaches often leverage motivational interviewing techniques to augment motivation to change drug use habits [20]. Motivation to change use habits reportedly represents a major barrier to successful cannabis intervention implementation for young adults with FEP [18]. Harm reduction approaches, especially when using motivational interviewing, may thus be well suited as an alternative to people who are ambivalent or reluctant to reduce their cannabis use but who may be ready to modify their cannabis use practices. Examples of such modifications might include reducing the number of times they use cannabis before work to avoid work problems, avoiding cannabis use while using other substances to reduce compounded deleterious physical or mental health harms, and so forth.

Although used to address problems related to other substances for many years [20], harm reduction initiatives for cannabis are only just emerging [21-23]; few trials (in nonclinical populations [24]) have evaluated their effectiveness, but initial findings are promising. However, there are, so far, no cannabis harm reduction interventions that have been specifically designed and evaluated for people with FEP who use cannabis [8]. This population is characterized by distinct clinical features, such as impaired attention and memory, and cognitive deficits [25], which must be considered when designing or recommending interventions. More research is needed to evaluate whether harm reduction interventions could benefit people with FEP and continued cannabis use, who are at risk of experiencing related harms.

### Study Aims

Our team developed a novel harm reduction intervention for individuals with FEP who are interested in reducing their cannabis use–related harms, called the Cannabis Harm–reducing Application to Manage Practices Safely (CHAMPS), which is delivered through a smartphone app. The development of CHAMPS was informed by related projects in cannabis use and psychosis conducted by our team [8,18,26] and co-designed in collaboration with clinicians, patient-partners, and experts in cannabis and psychosis (see intervention description in the Study Arms section). To our knowledge, the CHAMPS intervention represents the first psychosocial mobile health intervention aiming to reduce cannabis use–related harms in people with FEP. Our team is also currently working on a psychosocial mobile health intervention that addresses CUD in people with FEP and focuses on decreasing cannabis use or cessation [27].

Due to the paucity of cannabis harm reduction research in this population, a preliminary assessment of trial feasibility and intervention acceptability as well as an initial assessment of key clinical outcomes were necessary before conducting a large-scale randomized controlled trial (RCT) of CHAMPS [28]. As EIS represent the mainstay of treatment for FEP [29], we opted to evaluate this novel intervention as an adjunctive clinical tool to existing services. We will thus evaluate CHAMPS+EIS versus EIS-only in a multicenter, 2-arm, parallel group, open-label, randomized pilot study. The primary objectives of our study are to assess whether it is feasible to conduct a future RCT of the CHAMPS intervention in people with FEP who use cannabis and want to modify their cannabis behaviors and determine if CHAMPS is an acceptable intervention for this population. Secondary objectives aim to descriptively determine whether CHAMPS+EIS influences the use of protective behavioral strategies (PBS) and motivation to change PBS, compared to EIS only, in young people with psychosis who use cannabis. Exploratory objectives aim to descriptively estimate the effect of the intervention arm on cannabis-related problems, quality of life, cannabis and other substance use, severity of dependence, psychotic symptoms, and health care service use. We hypothesize that CHAMPS will be acceptable to those randomized to the CHAMPS+EIS intervention arm and that the pilot trial will demonstrate adequate feasibility, with a minimum trial retention rate of 60%.

## Methods

### Participants and Recruitment

This study will involve 100 young adults (50 per arm) who meet the study’s eligibility criteria. Briefly, the inclusion criteria are having a diagnosis of psychosis, being 18 to 35 years old, having recent cannabis use, and being treated at an EIS (see Textbox 1 for detailed inclusion and exclusion criteria). Participants will continue their usual pharmacological and psychosocial interventions offered at the EIS, including taking any medication prescribed for their psychosis or other health concerns while participating in this study; these will be recorded in the patient study file.

**Figure 1:**
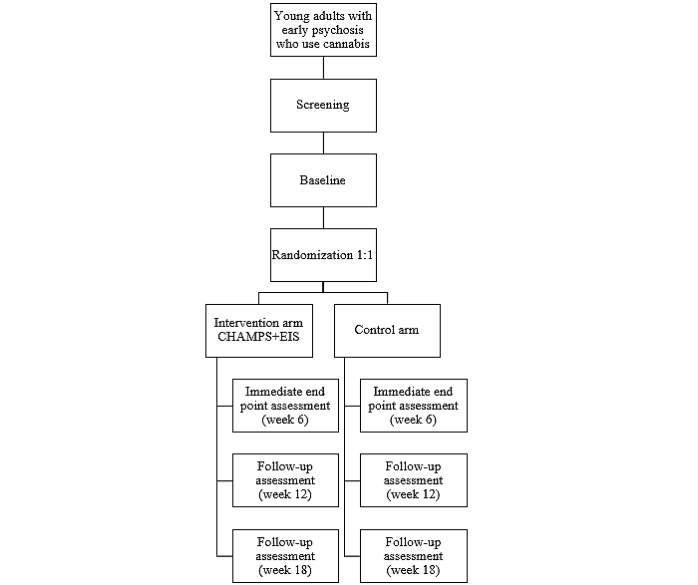
Study flowchart. CHAMPS: Cannabis Harm-reducing Application to Manage Practices Safely; EIS: early intervention service.

If the CHAMPS intervention is considered clinically relevant for a potential participant, a clinician approaches this person, briefly explains the intervention, and asks about any interest in meeting the research team to learn more about the trial. If the person agrees, the referring psychiatrist or clinician collects screening and medical eligibility information. In other words, participant recruitment will use purposive sampling. The research staff then schedules a full screening visit, where eligibility is confirmed, and informed consent is obtained from interested participants (Figure 1; Multimedia Appendices 1 and 2). For study visits conducted via telepsychiatry, e-consent and verification of comprehension are documented using the Research Electronic Data Capture (REDCap; Vanderbilt University) web-based data entry system [30].

Clinicians will assist participants as needed, as they download the CHAMPS app from the Apple Store or the Google Play Store. If potential participants do not own a smartphone, the research staff will loan them a smartphone for the study duration. All participants will receive a compensation of CAD $30 (US $22.14) for participating at each visit (eg, screening, baseline, and follow-up assessment visits), with a maximum of CAD $150 (US $110.69) per participant. In addition, participants randomized to the EIS-only arm are offered CHAMPS access after their trial completion; these data will not be collected for research purposes.

**Inclusion and exclusion criteria for participants and recruitment sites.**

**Inclusion criteria**

- Participants
  Young adults (18 to 35 years of age)
  Diagnosis of a psychotic disorder (including primary psychotic disorders, mood disorders with psychotic features, substance-induced psychosis, etc)
  Receiving care for psychosis at early intervention services for at least 2 months
  Recent cannabis use (in the last 30 days)
  Interest in changing cannabis-related practices
  Provide informed consent
  Comply with study procedures
  Read French or English
- Recruitment sites
  Specialized early intervention services for the treatment of people with early-phase psychosis
  Demonstrate capacity to provide early intervention services for psychosis
  Agree to offer Cannabis Harm–reducing Application to Manage Practices Safely alongside early intervention services
  Have sufficient patients to achieve enrollment targets
  Have a sufficient psychiatrist and clinician availability and acceptance of the study to refer patients
  Adhere to trial procedures

**Exclusion criteria for participants**

- Participation in another cannabis-focused intervention or treatment
- Currently seeks cannabis use disorder treatment

The lead site is located at the Centre Hospitalier de l’Université de Montréal (CHUM). Recruitment takes place at 6 EIS clinics in Nova Scotia and Quebec, Canada, which have varying clinical and sociogeographical contexts. See Textbox 1 for inclusion criteria for EIS. Each local site has a similar structure that includes clinicians (eg, case workers and psychiatrists), research staff, a research coordinator, and site principal investigators (PIs). With the assistance of local site PIs, our research team conducts a project presentation for clinicians working with this population at collaborating sites. Clinicians who are available to recruit and can adhere to CHAMPS trial procedures and requirements recruit participants for this study. Local site investigators responsible for local study oversight are invited as study coauthors. Clinicians contribute to recruitment and support using the CHAMPS app. Research staff and coordinators contribute to data collection, assist with recruitment, and conduct follow-up assessments. Local site PIs oversee research ethics board processes, recruitment, staff training, and coordination with the lead site. The lead site additionally oversees any potential study implementation challenges, ensures the quality of the data collection and databases, and monitors study progress.

### Randomization and Blinding

Participants are randomized in a 1:1 ratio to either arm (EIS-only and EIS+CHAMPS). Randomization is stratified by sex and CUD status using random permuted blocks. The CHUM Center for Integration and Analysis of Medical Data generates the allocation sequence. Access to the randomization code is strictly controlled by the data management team. Individual participant codes can be broken without unblinding the sequence allocation of other participants. Due to the nature of the intervention, clinicians and research staff collecting data cannot be blinded; however, the research staff conducting data analysis are blinded to group assignment. Participants are not able to switch arm assignments.

### Study Arms

EIS-only (ie, “Control”) offers EIS to all participants as per the standard of care at participating sites. Many EIS international and national guidelines are available and typically followed by these programs [31-33]. Most EIS offer similar services, including integrated treatments such as psychopharmacology and psychosocial interventions (eg, case management, family interventions, cognitive behavioral therapy, motivational interviewing, vocational recovery interventions, and outreach interventions) [29]. Regarding substance use services, a survey found that the majority of English-speaking Canadian EIS reported offering informal and general substance use services to patients (eg, individual psychoeducation), and only 12% reported offering cannabis-specific formal interventions [34]. A similar survey of 28 of 33 EIS in Quebec found that only 16 of 28 EIS offered group or individual substance use services [35]. The Quebec EIS framework explicitly states that substance use is important to address, ideally using motivational interviewing and cognitive behavioral strategies, although no formal harm reduction–focused interventions are recommended [33]. Practices may differ between EIS across Canada; we can expect at least some EIS to offer an unstructured, nonspecific, informal substance use intervention. Most EIS use a case management model, where a clinician takes the central role of treating a patient [29]. Any visits and services offered (eg, in person or via teleconsultation) at EIS will be considered “usual care.”

CHAMPS+EIS (ie, “Intervention”) arm offers participants access to EIS usual care in addition to the CHAMPS intervention. CHAMPS is a psychosocial intervention that aims to promote positive behavioral changes and a reduction of cannabis use–related harms, using motivational interviewing, psychoeducation, skills training, and personalized feedback strategies. CHAMPS is self-guided and delivered through a smartphone app; this delivery mode was guided by previous studies signaling an acceptability of and preference for technology-based interventions [36,37]. The CHAMPS protocol recommends accompanying clinicians to provide support using CHAMPS if participants request it as well as to encourage its use. If the participant consents, accompanying clinicians can also access the participant dashboard, which shows high-level app data, such as how many modules they have completed, as well as participant responses in the CHAMPS intervention (eg, goals they have set during module 4, reasons for changing cannabis use stated in module 2). No additional participant data will be shared with accompanying clinicians.

**Figure 2:**
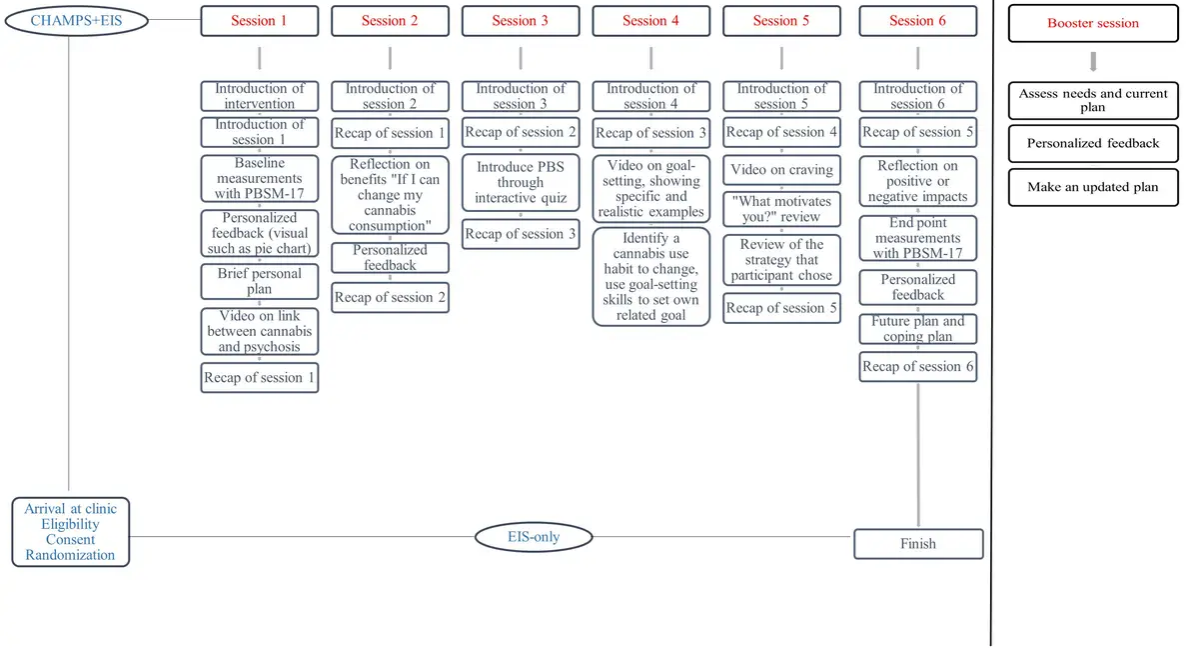
CHAMPS intervention detailed description. CHAMPS: Cannabis Harm-reducing Application to Manage Practices Safely; EIS: early intervention service.

There are 6 modules in the intervention and a booster session module 4 weeks after the last module (see Figure 2). Each module is expected to take up to 15-20 minutes to complete. In essence, CHAMPS informs participants about cannabis use and its effects on people with FEP, offers strategies to reduce related harms (inspired by harm reduction tools), and guides participants to set their own cannabis harm reduction-related goals to help improve their well-being. Suggested goals can target participants’ physical or mental well-being, social relationships, or even cannabis use itself; however, no goal will be imposed, and participants will decide which goals they would like to work on. Table 1 provides a detailed description of each module, and Textbox 2 provides examples of harm reduction strategies and goals from CHAMPS.

**Table 1:** Detailed CHAMPS^a^ module descriptions.

Week	Module	Description
1	Module 1	Measures baseline cannabis protective behavioral strategies (PBS^b^) and automatically provides participants with personalized feedback on their cannabis practices, as well as suggestions on what cannabis practices they could improve
2	Module 2	Explores what is important to participants and the possible benefits of adopting safer cannabis use strategies
3	Module 3	Exposes participants to various evidence-based cannabis PBS using interactive psychoeducational techniques
4	Module 4	Introduces participants to goal setting and asks them to set their own cannabis-focused goals, which they can immediately implement in their lives
5	Module 5	Helps participants reflect on their goal progress and offers help to the participant in either revising their current goal or adopting a new goal
6	Module 6	Reassesses cannabis PBS and encourages participants to continue improving their cannabis practices
10	Booster session	Four weeks postintervention, participants will receive a booster session, also lasting between 15 and 20 minutes. The purpose of this booster session will be to review the main components of the intervention, remind participants of what they explored about their cannabis use as well as goal setting during the intervention, and revise it as needed

^a^CHAMPS: Cannabis Harm-reducing Application to Manage Practices Safely.

^b^PBS: protective behavioral strategies.

**CHAMPS harm reduction strategies and sample goals.**

**Limit the amount of cannabis you smoke in one sitting**

“I will put a quarter of the amount of cannabis I usually put in my joints for a prespecified period of time.”

**Avoid mixing cannabis with other drugs**

“I will avoid using alcohol and cannabis at same time during a pre-specified period of time”

**Avoid using cannabis to help cope with emotions such as sadness, depression, anxiety, and fear**

“Instead of using cannabis, I will practice using alternate and healthier coping strategies (eg, trying a new sport, calling a friend) the next time I feel negative emotions”

### CHAMPS Intervention Development

The CHAMPS intervention was inspired by the Behavioral Change Wheel theoretical framework, which has been used to design behavioral interventions in various settings including for young people at ultra-high risk for psychosis [38,39]. The CHAMPS intervention uses the following behavior change techniques detailed in this framework: feedback on behavior, brief action planning, modeling of behavior, reflection of health, social and environmental consequences, instructions on how to perform a behavior and behavioral experiments, review behavioral goals, and behavioral rehearsal and practice. The findings of our qualitative study outlining barriers and facilitators in a variety of cannabis-focused interventions according to young adults with FEP who use cannabis and to accompanying clinicians also helped guide the CHAMPS intervention development [18]. This study supported the use of harm reduction for people who used cannabis and lacked motivation for more intensive cannabis-focused treatments and reported openness to technology-based interventions, citing potential benefits such as quicker intervention access for patients, decreased clinician workload, and so forth. The modalities of CHAMPS were informed by our preference study for cannabis harm reduction interventions, which surveyed 89 young adults with FEP using cannabis. This survey, which featured a quantitative discrete choice experiment design, found that respondents significantly preferred interventions with shorter durations and sessions, technology-based interventions, and brief booster sessions (personal communication by SC-M and DJ-A, September 2023 on patient preference survey results; associated manuscript submitted for publication).

**Figure 3:**
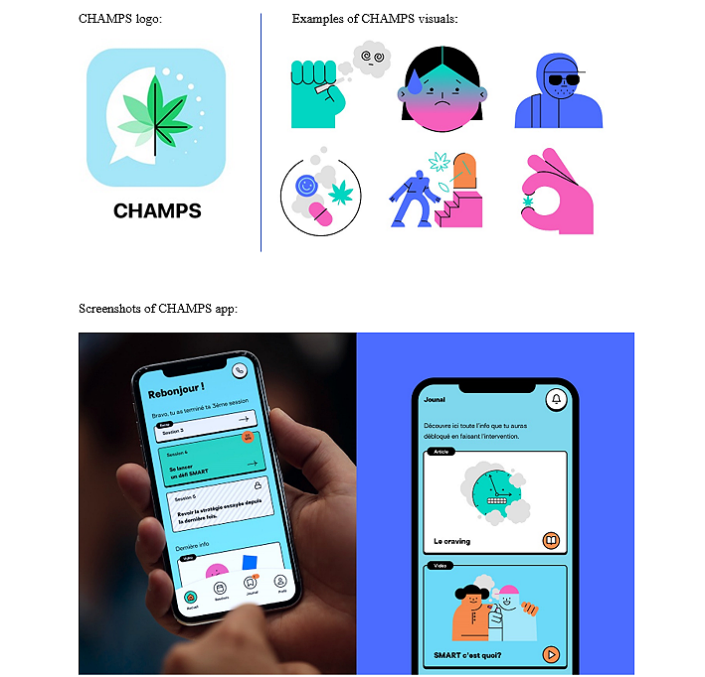
Examples of CHAMPS app. CHAMPS: Cannabis Harm-reducing Application to Manage Practices Safely.

The design of CHAMPS used a participatory approach, where people with lived experience were integrated into different facets of the study. We created the CHAMPS patient advisory committee comprising young individuals who used cannabis, had FEP, and attended EIS. This committee consulted on and helped co-design CHAMPS. For example, this committee contributed to the selection of the visuals we used, the tones and language of our CHAMPS content, the phrasing, the frequency and content of add-ons such as notifications, and so forth (see Figure 3 for examples of CHAMPS app visuals). Some patient-partners were also involved in the content creation, as they coproduced video testimonials of their experience with changing their cannabis use habits. Additionally, 1 patient-partner was involved in knowledge dissemination related to this project, copresenting on the protocol at a scientific conference and detailing the involvement of the patient advisory committee.

### Outcomes and Data Collection

#### Participant Characteristics

Sociodemographic data are collected at baseline using a questionnaire on sociodemographic outcomes that include gender identity, sex, ethnicity, educational attainment, vocational status (eg, employment), income, and housing status.

#### Primary Outcomes

The feasibility of conducting a future RCT will be determined through the trial’s retention rate. The retention rate will be calculated by dividing the number of participants who completed all end point assessments (week 6) by the number of total randomized participants at baseline. As discussed, there are so far no other harm reduction–oriented studies in this population to guide our study design. Therefore, to establish an adequate minimum retention rate, we looked at related trials of (1) psychosocial mobile health interventions in populations with psychotic disorders, which had retention rates ranging from 76% to 100% [40-43] and (2) RCTs of face-to-face cannabis interventions for people with FEP, which had retention rates ranging from 52% to 96% [44-47]. The mobile health intervention trials either did not include comparison groups, did not focus specifically on individuals with FEP who used cannabis, or did not evaluate cannabis-focused interventions. The face-to-face interventions all focused on cannabis use in people with psychosis, but the delivery method precluded generalization to our trial. Given our intervention characteristics, we expect our retention rate will be on the lower end of these spectrums. We believe a 75% retention rate is a conservative estimate, but we are setting the minimum threshold for retention rate at 60%, which we believe represents a clinically important retention rate for our population.

Acceptability of the CHAMPS intervention will be assessed using the CHAMPS completion rate. This rate will be calculated by dividing the number of participants who completed at least the first 4 modules of CHAMPS by the number of total participants randomized to CHAMPS. We selected this completion threshold as the first 4 modules represent the core of the intervention, exposing participants to psychoeducation, motivational interviewing, personalized feedback, and skills training.

#### Secondary Outcomes

The efficacy of CHAMPS will be described by examining the change in participants’ use of PBS, using the Short Form Protective Behavioral Strategies-Marijuana measure [21]. This is a 17-item self-report measure that includes evidence-based strategies that promote safer cannabis experiences. The marginal reliability of the short-form measure is high (0.93) [21].

The efficacy of CHAMPS will also be estimated by measuring the change in participants’ motivation to change PBS, which is assessed using the Readiness to Change Questionnaire (RCQ) modified for cannabis use [48,49]. The RCQ is a 12-item clinician-administered measure based on Prochaska and DiClemente stages-of-change model and assesses 3 stages of change: precontemplation, contemplation, and action [49]. The internal consistency is satisfactory; the 3 stages of change scales have acceptable internal consistency, ranging from Cronbach α=.73 to α=.85 [48].

#### Exploratory Outcomes

Cannabis use–related physical, psychological, social, and functioning problems will be assessed using the Marijuana Problems Scale, a 19-item self-report instrument that measures changes in problems related to cannabis use over time [50,51].

Cannabis and other drug use in the past 2 weeks will be assessed using the Timeline Follow-back. This tool has high test-retest reliability and has been validated using other measures of drug-related problems or in clinical populations [52,53]. This tool has previously been used in a trial of people with psychosis who use cannabis [54]. This tool assesses the self-reported days of cannabis use per week, as well as the type of cannabis products, quantity, and dose or concentration of cannabinoids. The main end point for this outcome will be the number of days per week of cannabis use. The frequency of other drug use in the past 2 weeks will also be assessed using this tool.

Psychotic symptoms will be measured using the Positive and Negative Syndrome Scale-6, which is easily administered and has good psychometric properties [55,56]. The scale consists of 6 items: delusions, conceptual disorganization, hallucinatory behavior, blunted affect, passive or apathetic social withdrawal and lack of spontaneity, and flow of conversation.

Severity of cannabis dependence will be measured using the Severity of Dependence Scale [57]. This is a short, easily self-administered instrument developed to provide a brief measure of the psychological aspects of dependence experienced by users of various types of illicit drugs. It is a measure of compulsive use. This scale has good internal consistency (Cronbach α ranging from .80 to .90) [57,58] and has previously been used in trials with people with FEP [5,59].

Health service use data are collected via participant self-report and are confirmed as needed through patient medical record abstraction and contact with the clinician, gathering information on the total number of past-month emergency visits and days of hospitalization.

Data relating to the reception of EIS, CHAMPS usage data, and participant satisfaction will be used to complement data on CHAMPS acceptability and trial feasibility. We collect data on participants referred, screened, considered eligible, recruited, and consented; participants who initiated the intervention; and participants who completed baseline assessments. We will calculate the control group completion rate by dividing the total participants randomized to the control group by the number of participants who, at week 12, are still participating in EIS treatment.

Understanding of CHAMPS acceptability by study participants will be complemented by an evaluation of participant satisfaction with CHAMPS, using the Client Satisfaction Questionnaire-I, which is a modified version of the Client Satisfaction Questionnaire-8 and which measures global satisfaction with a web-based intervention [60]. This measure has an adequate internal consistency, as represented by Cronbach α=.93 for the 8-item scale [60,61]. Those randomized to EIS-only will receive the Client Satisfaction Questionnaire-8 and accompanying instructions to evaluate the cannabis-related treatment components of EIS using this questionnaire.

### Data Collection and Management

Participation in the trial lasts up to 22 weeks, including up to 4 weeks between screening and randomization, 6 weeks of active CHAMPS intervention (immediate end point assessment), a booster session 4 weeks after the end of active CHAMPS intervention, and follow-up assessments conducted at weeks 12 and 18 (Table 2). Assessments are conducted either in person, over the phone, or using videoconferencing platforms. Communication and contact can be done digitally, over the telephone, or during in-person visits (only if confidentiality can be strictly maintained). All interactions are recorded in the patient study file. Furthermore, sites track participant interactions and collect information on recruitment, ineligibility reasons, and nonparticipation reasons. The research team uses these data to refine the recruitment strategy during the trial if needed.

**Table 2:** Schedule of assessments.

Assessment name	Time point										
	Screening visit (week-4-2)	Baseline (W-2-W0)	Week 1	Week 2	Week 3	Week 4	Week 5	Week 6	Week 10	Week 12	Week 18
Screening form	✓										
Informed consent form	✓										
Locator form	✓							✓		✓	
Demographic questionnaire		✓									
Social Provision Scale-10		✓									
Readiness to Change Questionnaire		✓						✓		✓	✓
Marijuana Problems scale		✓						✓		✓	✓
Timeline Follow-back		✓						✓		✓	✓
Positive and Negative Syndrome Scale		✓						✓		✓	✓
Severity of Dependence Scale		✓						✓		✓	✓
Health care use		✓						✓		✓	✓
Randomization		✓									
Protective Behavioral Strategies—Marijuana		✓						✓		✓	✓
Client Satisfaction Questionnaire								✓		✓	
Adverse events								✓		✓	✓
Intervention and services form								✓		✓	✓

Data are entered into REDCap by site research staff or directly by the participant (through the CHAMPS app). The data dictionary will be built on REDCap and will be available upon request to the corresponding author. Final data quality assurance checks will be conducted by the data management team, and the study database will be locked, prohibiting further modification. The REDCap system was developed and implemented by the Center for Integration and Analysis of Medical Data to ensure that guidelines and regulations surrounding the use of computerized systems in clinical trials are upheld.

Participants are attributed a unique numeric study identifier to anonymize data, and data are password-protected on computerized databases and kept strictly confidential. Records are accessible only to research staff. Intervention arm participants are provided with detailed information regarding their data usage and protection (Multimedia Appendix 3). The final analysis data set will be kept on the secure server at the CHUM in Montreal, Québec, for 10 years, in line with *Fonds de la recherche en santé du Quebec* and CHUM research ethics board requirements.

An independent data and safety monitoring board examines accumulating data to ensure the protection of participants’ safety during the study. This board determines whether there is ground for trial continuation; evidence that study procedures require modifications; or whether the trial should be halted for participant safety, intervention efficacy issues, or inadequate trial performance reasons.

### Sample Size

**Figure 4:**
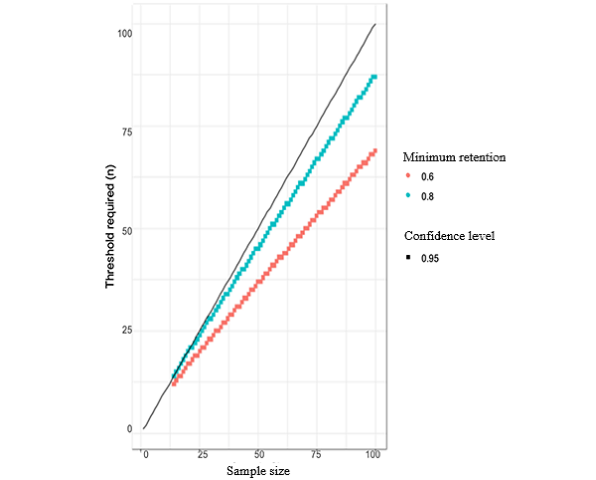
Diagram of trial retention rates.

The sample size for pilot trials is typically not driven by power calculations but rather by convenience or precision estimates [62], both of which we used to inform our sample size. Using a precision-based approach, we estimated the number of participants we would need to retain in this trial to detect with 95% confidence the true retention rate for a future trial. As aforementioned, based on related literature and our current context, we deemed 60% a clinically significant retention rate for this trial [40-43]. As seen in Figure 4, if we recruit 100 participants and retain at least 69 participants at the end of the study, we would have 95% confidence that the true (ie, population-level) retention rate is above 60%. Regarding convenience, our existing partnerships with Canadian EIS clinics and experience recruiting relatively large samples from this population [63] lead us to believe that recruiting 100 eligible individuals is feasible for this trial.

### Statistical Analysis

We will be analyzing primary outcomes using univariate analyses. The trial retention rate will be measured in terms of the percentage of randomized individuals retained in the intervention at end point assessment and will include a 1-sided 95% confidence interval to estimate the lower bound of the retention rate. CHAMPS acceptability will be reported using univariate analysis. We will also conduct exploratory analyses to test whether belonging to either study arm influenced EIS completion rates.

To describe sociodemographic and clinical variables at baseline, trial parameter data, and secondary and exploratory outcomes, we will conduct univariate analyses. We will use multivariable statistical methods to explore the following outcomes: participant satisfaction, use of cannabis PBS, and motivation to change cannabis PBS. To compare satisfaction between groups, we will use a hierarchical linear regression. The first regression model will insert the intervention arm as the independent study variable; the second model will insert other covariates. We will also conduct a hierarchical linear regression exploring the use of cannabis PBS over time as the outcome; the first regression model will insert study arms as the independent variable, and the second model will insert baseline PBS scores and other relevant covariates. To look at motivation to change, analyses will be done as with cannabis PBS, using hierarchical models that look at RCQ scores as the outcome and control for RCQ scores at baseline.

In multivariable models, we will include sites as random effects and will include certain covariates of interest that differ at baseline. These covariates may include age, sex, gender, study site, education status, employment, housing status, social support, and baseline clinical characteristics including cannabis use and CUD severity. Sensitivity analyses will explore key outcomes in subgroups of participants (eg, location-based subgroups), as well as the use of different thresholds to determine whether participants have completed CHAMPS.

Missing data will be handled issuing guidance of the missing data treatment for each instrument. The proposed analyses will include all participants with at least 1 nonmissing visit. Our analysis plan includes both intention-to-treat and per-protocol analyses.

### Ethical Considerations

Ethical approval for the proposed study was obtained from the CHUM research ethics board (#20.433). Prior to initiating the study, local approval to conduct the study at each respective location was also obtained. This study will be conducted in accordance with the ethical principles outlined in the Declaration of Helsinki, the International Conference on Harmonization Good Clinical Practice Guidelines, and all other applicable regulatory requirements. This pilot trial protocol was developed in accordance with the extension of the Consolidated Standards of Reporting Trials statement for randomized pilot or feasibility trials [64] (Multimedia Appendix 4).

Site PIs will ensure that the trial is conducted according to good clinical practice guidelines and may perform quality assurance audits for protocol compliance. The local research ethics board may inspect research records for verification of data, compliance with federal, provincial, and local guidelines on human participant research and to assess participant safety. Adverse events reported by the participant will be recorded electronically by study staff and reported to the site PI, study nurse, and study clinician for assessment and referral as appropriate. Site PIs will review each adverse event and assess the possible relatedness of any adverse event to the study intervention or other study procedures. Each of the sites has established practices for managing medical and psychiatric emergencies, and the study staff will use these procedures.

## Results

Enrollment took place from December 2021 to June 2023. Data collection and analysis are expected to be completed by early 2024, and study results will be submitted for publication in peer-reviewed scientific journals thereafter. For participants who opt to receive the results, we will apply various knowledge transfer tools (eg, health cards, summaries with charts and graphics, video clips accessible on the web or through social media) in English and French to effectively translate our findings. Furthermore, we will leverage our networks to relay study findings in a format that is tailored to this population. We have designed an ancillary qualitative study that will explore CHAMPS pilot trial participants’ and accompanying clinicians’ experiences regarding the CHAMPS intervention and its implementation in EIS. This ancillary study will serve to further understand the barriers and facilitators of CHAMPS and its implementation and can inform future trials of similar interventions.

## Discussion

### Principal Findings

This mobile health and harm reduction intervention is the first of its kind to be evaluated in the context of reducing cannabis use–related harms for this population. We consider that CHAMPS was strengthened by its unique development process, using preference research to shape the intervention and involving individuals with lived experience as CHAMPS coproducers. Despite potential barriers to coproduction research, such as a high turnover rate of clinicians or researchers and stigma toward patients [65], our patient-partners anecdotally reported that the coproduction process was successful in building healthy relationships between coresearchers and patient-partners and provided opportunities for personal growth. Feedback from our co-researchers also highlighted the benefits, including the production of a well-informed intervention that valued the knowledge of all contributors.

Another strength of this pilot trial is its rigorous and carefully crafted design, considering the limited literature on cannabis use–related harm reduction in people experiencing FEP and the use of mobile health technology in this context. The CHAMPS trial design, as a multicenter, randomized pilot study, will enable rigorous assessment of the feasibility of running a full-scale RCT with CHAMPS and of the acceptability of the CHAMPS intervention for this population. Furthermore, we are collecting data exploring how CHAMPS may potentially influence harm-related outcomes. The results of this pilot trial and its ancillary qualitative study may offer valuable insights for future trials assessing similar psychosocial interventions.

Mobile health interventions like CHAMPS may contribute to the alleviation of strained health care systems by improving accessibility for participants and providing an independent tool to aid clinicians in delivering optimal care to their patients [66]. Surveys and personal communication by SC-M and DJ-A on September 2023 on patient preference survey results (associated manuscript submitted for publication) demonstrate the theoretical acceptability of and preference for mobile health interventions to target CUD or cannabis use by people with FEP [36,37]. However, the implementation of a novel mobile health intervention or concerns around data confidentiality may hinder the potential acceptability of this intervention. More research piloting the use of new technologies, such as CHAMPS, to address existing mental health challenges in individuals with FEP needs to be undertaken before recommending these initiatives in clinical contexts.

### Limitations

The present trial has limitations and may face a variety of possible challenges. Previous data suggest that continued cannabis use predicts persistently poor treatment adherence for people with psychosis [67]; if such patterns also translate to psychosocial intervention adherence, then this may lead to retention challenges for our trial. We are recruiting participants from Quebec and Nova Scotia provinces, which differ in their sociocultural and clinical contexts from each other and other provinces. To explore the potential impact of these differences, we will be doing sensitivity analyses to explore possible site-specific effects on outcomes. On the other hand, our potentially heterogeneous sample may provide researchers with insights regarding the effectiveness of CHAMPS in multiple real-world contexts, facilitating generalization to other settings. The context-specific adaptations that occurred as a result of the ongoing COVID-19 pandemic (eg, transitioning to telehealth services) and which our participants may have experienced may influence the results of our study. For example, tightening restrictions may have resulted in less frequent in-person clinical consultations. These tightened restrictions may also have particularly impacted people with psychosis, who are likely to be single and have limited social networks [68], and resulted in higher levels of isolation and emotional distress for certain participants. It is possible that these changing regulations and societal restrictions may have influenced the reception, effectiveness, satisfaction, or other related outcomes of our intervention and EIS. The role of this particular context must be considered during our analyses and may limit the generalizability of our study findings. Finally, while we consulted the literature to determine best practices for data protection and confidentiality strategies while designing CHAMPS, concerns around data protection and privacy have previously been a hindrance to the acceptance of mental health–related apps [66]. It is thus still possible that we will face confidentiality and privacy issues or that risk perception by participants may impact our capacity to recruit and engage them in the intervention.

### Conclusions

CHAMPS uniquely combines evidence-based approaches, harm reduction strategies, patient perspectives, and mobile health technology to address cannabis use–related harms in young people with FEP and persistent cannabis use, potentially addressing an unmet need in this population. Our study findings have the potential to advance knowledge in the cannabis and psychosis fields. To gain a better understanding of harm reduction interventions, future research should prioritize conducting robust, large-scale RCTs. Preliminary studies, like the present pilot trial, are urgently required to refine optimal design and approaches for these interventions.

## Appendix

Multimedia Appendix 1 Example informed consent form.

Multimedia Appendix 2 CHAMPS screening form.

Multimedia Appendix 3 CHAMPS data privacy statement.

Multimedia Appendix 4 CONSORT checklist for pilot or feasibility trial [65].

## Abbreviations

CHAMPS: Cannabis Harm-reducing Application to Manage Practices Safely
CHUM: Centre Hospitalier de l’Université de Montréal
CUD: cannabis use disorder
EIS: early intervention service
FEP: first-episode psychosis
PBS: protective behavioral strategy
PI: principal investigator
RCQ: Readiness to Change questionnaire
RCT: randomized controlled trial
REDCap: Research Electronic Data Capture
