# Supplementary material for: Evaluation of a Cannabis Harm Reduction Intervention for People With First-Episode Psychosis: Protocol for a Pilot Multicentric Randomized Trial
Source: JMIR Res Protoc. 2023 Dec 18;12:e53094. doi: 10.2196/53094 (PMC10758938; doi:10.2196/53094)
Supplement: Multimedia Appendix 1 [file resprot_v12i1e53094_app1.docx]

INFORMATION AND CONSENT FORM

Project title: A randomized pilot trial of a cannabis harm reduction e-intervention (CHAMPS) for young adults with early psychosis who use cannabis

Principal investigator at CHUM: Dr. Didier Jutras-Aswad, Psychiatrist, Addiction Psychiatry Unit, CHUM

Co-investigators CHUM at CHUM: Dr. Amal A-Baki, MD, Msc., FRCPC, JAP (CHUM)

Tania Lecomte, PhD, IUSMM - Clinique Connec-T (ISMM du CIUSSS de l’Est de Montréal)

Dr. Marc-André Roy MD FRCP, Clinique Notre-Dame des Victoires (ISMQ, CIUSSS de la Capitale-Nationale)

Co-investigators out of CHUM

(out of Quebec): Dr. Philip Tibbo, MD FRCPC, Nova Scotia Early Psychosis 
Program (NSEPP), Halifax

Dr. David Crockford, MD, FRCPC, Foothills Medical Centre Early Psychosis Intervention Program (FMC-EPIP), Calgary

Funding Agencies: Direction Générale de la Santé Publique (DGSP)

Ministère de la Santé et des Services Sociaux

No of the multicentric project: MP-02-2021-9495

No of the project at the CHUM: 20.4333

PREAMBLE

You are being invited to take part in a research project because you have been diagnosed with a psychotic disorder, you are currently using cannabis and you are open to changing your cannabis practices. Before agreeing to take part in this project and signing this informed consent form, please take your time to read and carefully consider the following information.

This form may contain terms which you do not understand. Please ask a research team member any questions you feel might be helpful and to explain any word or information which is not clear to you.

NATURE AND OBJECTIVES OF THE STUDY

In Canada and elsewhere, early intervention services (EIS

) are the usual treatment for people with early psychosis. These services target youth during what is usually called the “critical period” (first two to five years) following the onset of a psychotic disorder, during which time certain interventions can help improve health compared to usual care. There are only few interventions or services that try to prevent cannabis use or cannabis-related harms from becoming a problem in people with psychosis who continue to use cannabis. That is why our team developed an intervention called Cannabis Harm-reducing App to Managing Practices Safely (CHAMPS).

To our knowledge, CHAMPS is the first psychosocial intervention that is delivered through your phone, which was designed to help reduce cannabis-related harms in young people with early psychosis who use cannabis. Our aim is to find out if this intervention works for youth who use cannabis.

The “CHAMPS” App proposes 17 strategies to reduce cannabis harms and offers tools to help you reach your consumption goals.

The main thing that researchers are trying to do is to assess whether the intervention works, gather your experience using a phone application for receiving services, and whether there is any change in your cannabis use or health through participating in this intervention.

NUMBER OF STUDY PARTICIPANTS AND LENGTH OF THE PARTICIPATION

A total of 100 participants will be recruited into this study, which will be carried out at 5 clinics across Canada including 30 participants from the CHUM. Your participation in this study will last up to 22 weeks and the project will last for 2 years.

NATURE OF PARTICIPATION REQUESTED

If you are eligible and you agree to participate in this study, after signing this consent form you will be asked to comeback 3 to 4 times to the clinic to complete. During the intervention period which will last for 6 weeks, you will be requested to complete depending on the group you will be randomized in:

- Either the e-intervention using your smartphone and services you usually receive at the clinic (Intervention group).
- Or services you’ll receive as usual at the clinic (EIS only group).

Any visits and services offered in the EIS arm will be considered "usual care” and administered either through in-person clinic visits, phone calls, or video calls. Relevant service information will be collected for study purposes.

If you are randomized to the intervention arm, your case manager and doctor from the clinic will only see if you have used the e-intervention and complete the modules, they will not have access to the content and what you write. However, if the staff notices that there is information that you are recoding in the app that may be important for your safety or the safety of others, information may be passed along to your doctor or case manager.

CONDUCT OF THE STUDY/PROCEDURES

The study will include three phases:

1. **Screening period**
2. **Intervention period in 2 phases**
   1. Baseline visit : duration approx. 1h
      1. Complete questionnaires (2-week period)
      2. Randomization

You will be randomly assigned (like flipping a coin) to one of the 2 groups:

- Group (1): CHAMPS + EIS (usual care)

- Group (2): EIS only (usual care only)

- - 1. Completion of the questionnaire: “Protective behavioral strategies – Marijuana” (PBS-M)
- For the intervention group (1): this questionnaire will be completed on your smartphone after installing the app and starting Module 1.
- For the control group (2): this questionnaire will be completing online like all the other questionnaires.
  1. Intervention: For 6 weeks, and each module will last between 15-20 min.

1. Follow-up period: visit at 6, 12 and 18 weeks

- Booster session (only for CHAMPS + EIS group): at 4 weeks after you are done the app/intervention.

In order for the research team and your doctor to make sure that the study is a fit for you, some information about your medical history has already been confirmed. This information will be used for research purposes.

1. Screening phase: (duration approx.1-hour, to confirm eligibility: 2-week period including consent process and screening visit)

After the study team receives basic information about you to participate, and you still agree to take part in this study, the screening visit will be scheduled. During this visit the research staff will gather more information from you to make sure that the study will be a fit for you. Once we determine that you have completely understood your participation in the study, and provide your informed consent by signing this document your enrollment will be complete. After you sign the consent, you will also complete what is called a “Locator Form”. This form will include the best ways that the research staff can contact you for follow-up visits or to check in.

Every female participant will be asked if they are currently pregnant or nursing. If you are pregnant you will not be able to participate and if you become pregnant during the study, you will be withdrawn from the study. If you are withdrawn, you will continue to receive services as usual with your doctor.

If you choose to take part in the study your primary care physician/clinic will be notified of your participation. This process will be helpful if you wish to continue with your assigned treatment upon study completion. Prior to study completion, your study doctor will work with your clinical care team in order to avoid any disruption with your current treatment.

To obtain some health information and information about your social service use, we are asking your permission to access medical health record which contain some of your personal information.

1. Intervention phase
2. The questionnaires (see table 1. Schedule of assessments) administered throughout the study will ask questions about your drug and alcohol use, cravings, treatment services you have used and your feelings about those services, depression, anxiety, emotional well-being, quality of life, criminal activity, social and family history, medical and mental health. You can refuse to answer any question you do not want to answer.
3. Once screening assessments are administered and eligibility is confirmed, you will complete a set of questionnaires. Baseline assessments could take up to 1 hour to complete and may be done in-person or over the phone with the Research staff. Screening and baseline assessments can take place in the same day or separate days (within a week from screening depending on your availability and the availability of the research staff).
4. Randomization: You will then be randomly assigned to one of the groups:

- CHAMPS + EIS (intervention group + usual services)
- EIS alone (usual services)

If you are randomized to the “CHAMPS + EIS” group, the research staff will help you get set up with the app on your phone and you will start the first module together. This way the staff is there to help you get set up and answer questions. Please make sure you bring your phone to the baseline visit.

The Intervention

1. CHAMPS + EIS Group

If you are randomized to the “CHAMPS + EIS” group, you will download the app for your phone and complete 1 module per week. The research project will provide smartphones to people who do not have them for the duration of the project. There are 6 modules total. Each module will take about 15-20 min to complete. You will also continue to see your doctor or case worker as usual during this time for usual services. Below describes what each module is about.

- Module 1: collects information on strategies to manage your cannabis use, the app will give you with personalized feedback on your cannabis practices, as well as suggestions on how and what cannabis practices you could improve.
- Module 2:  explores your reasons for using cannabis and allows you to create your own plan to improve your cannabis practices.
- Module 3: you will be given some different strategies to manage your cannabis use using interactive and educational activities.
- Module 4: you are introduced to “goal setting” and are provided with the guidance and tools to set your own cannabis-focused goals, which you can start working toward.
- Module 5: feedback is given on the progress of your action plan, and help will be offered to either revise your current action plan or adopt a new action plan.
- Module 6: you will be encouraged to continue improving your cannabis practices as well as to gather information on your current cannabis practices.

Four weeks after you are done your modules, you will be offered an online booster session, also lasting between 15 and 20 min. The purpose of this booster session will be to review the main components of the intervention, remind you of the goal you set during the intervention, and to revise it as needed.

1. EIS (Early Intervention Services) only group

These services are offered as usual care at your clinic. Any visits and services offered in the “EIS only” group will be considered "usual care" and take place either through in-person clinic visits, phone calls, or video calls. Relevant service information including group intendance, intervention received, and services provided at your early intervention clinic will be collected by the research staff for study purposes.

After your participation to the study is complete (18 weeks), you will be offered access to the app free of charge. If you choose to use the app after you have completed 18-week participation, your consent would continue for 10 weeks until you have completed the application intervention. If you choose to have access to the intervention following the 18-week period, your total participation will be 28 weeks. In this case, you will be contacted at the 10-week timepoint to follow up on safety.

1. Follow-up visit: (duration approx. 30-45 min par visit)

Once the intervention complete, you will be asked to return to the clinic at 6, 12 and 18 weeks to complete a few additional questionnaires (see table 1. Schedule of assessments). We estimate that each follow-up visit will take between 30 and 45 min.

After those visits, your participation to the study will be completed.

YOUR RESPONSIBILITIES AND IMPORTANT PRECAUTIONS TO BE TAKEN

- By signing this consent form, you agree to follow the instructions given by your case manager, to go to the visits as scheduled in connection with the study and to submit to all assessments required as part of the study.
- You should inform as soon as possible your case manager, or a member of his team or the Research team, if you experience any adverse event that you think are related to the intervention, as it could affect your safety or health. Contact information is provided in the “Identification of contact people” clause.
- In case of emergency (evening, night, weekend, and holiday weekday), to report any adverse effect or injury related to the research, you must go to the emergency department of the closest hospital as needed and you will be seen by the doctor on call. You must mention that you are participating in this research project.

RISKS AND INCONVENIENCES

Participation in this study will expose you to the adverse event listed below. The research staff will discuss this with you. Other unexpected and sometimes serious adverse events may emerge. The research staff will monitor you at each visit to see if you have any adverse effect.

1. Inconvenient associated with the study procedures

Some of the questions asked may make you uncomfortable. You do not have to answer questions you do not wish to answer. If you do not understand a question, we will try to clarify it for you. If you get upset during one of the sessions, we will refer you to a specialized mental health professional. You may experience frustration because of all the testing and the considerable time investment the study demands.

2. Risks related to procreation

Person whose biological sex is female

Your participation in this research project may involve risks, known and unknown, to pregnant females, unborn children or breastfed infants. This is why pregnant and nursing females cannot participate in this study.

If your biological sex is female, you will be asked if you are pregnant throughout participation in the study. Also, if you are having sexual intercourse, you will be encouraged to use an acceptable method of contraception throughout their participation in the study.

Acceptable methods of contraception, from a medical point of view, are the following:

| 1. Oral contraceptives |
| --- |
| 1. Contraceptive patch |
| 1. Levonorgestrel implant |
| 1. Medroxyprogesterone acetate injection |
| 1. Intrauterine contraceptive device (IUD) |
| 1. Hormonal vaginal contraceptive ring |
| 1. Barrier (diaphragm or condom) |
| 1. Complete abstinence from sexual intercourse as life choice |
| 1. Surgical sterilization |

If you think you have become pregnant during your participation in this project, you will need to report immediately to your study doctor in order to discuss with him the different options. If you are pregnant, you will be removed from the study. If you agree, we will ask you to allow your treating doctor to forward to the study doctor and the funding agency the information about the follow-up of your pregnancy and your baby’s health at birth. You will need to sign a separate consent form.

BENEFITS

You may obtain a personal benefit from your participation in this study, but we cannot guarantee it. At the very least, the results obtained will contribute to the progress of the knowledge in this field.

CONFIDENTIALITY

During your participation in this study, the research team will collect and record information about you in a study file. They will only collect information required to meet the scientific goals of the study.

The study file may include information from your medical chart concerning your past and current state of health, your lifestyle, as well as the results of the tests, exams, and procedures that you will undergo during this research study. Your research file could also contain other information, such as your name, sex, date of birth and ethnic origin.

All the information collected during the research study will remain strictly confidential to the extent provided by law. You will only be identified by a code number. The key to the code linking your name to your study file will be kept by the Principal Investigator.

The study data will be stored for 10 years by the study doctor and the funding agency.

The study data may be published or shared during scientific discussions; however, it will not be possible to identify you.

For monitoring, control, safety, security, your study file as well as your medical charts may be examined by a person mandated by representatives, the institution, or the Research Ethics Board. All these individuals and organizations adhere to policies on confidentiality.

You have the right to consult your study file in order to verify the information gathered, and to have it corrected if necessary.

The study data may be published or shared during scientific discussions; however, it will not be possible to identify you.

COMMUNICATION OF OVERALL RESULTS

You can find out the overall results of this study if you ask the Principal Investigator at the end of the study.

POSSIBLE MARKETING

Your participation in this research project may lead to the creation of commercial products that may eventually be protected by a patent and/or other intellectual property rights. However, in this situation you will not be entitled to any financial compensation.

FUNDING OF THE PROJECT

The Principal Investigator and the institution received funding of the funding agency to carry out this research project.

COMPENSATION

You will receive $30.00 per scheduled visit, 5 visits are planned, for a total amount of $150.00 for costs and inconveniences incurred during this research study. For the screening visit, you will receive $30.00 for your time. If you withdraw from the study, or are withdrawn before it is completed, you will receive compensation proportional to the number of visits you have completed. If you do not have a smartphone, we will provide it to you for the duration of the study.

SHOULD YOU SUFFER ANY HARM

By agreeing to participate in this research project, you are not waiving any of your legal rights nor discharging the Principal Investigator, the funding agency, or the institution, of their civil and professional responsibilities.

VOLUNTARY PARTICIPATION AND THE RIGHT TO WITHDRAW

Your participation in this research project is voluntary. Therefore, you may refuse to participate. You may also withdraw from the project at any time, without giving any reason, by informing the research team.

Your decision not to participate in the study, or to withdraw from it, will have no impact on the quality of care and services to which you are otherwise entitled, or on your relationship with the clinical team providing them.

The study doctor, the Research Ethics Board, the funding agency may put an end to your participation without your consent. This may happen if new findings or information indicate that participation is no longer in your interest, if you do not follow study instructions, or if there are administrative reasons to terminate the project.

If you withdraw from the study or are withdrawn from the study, the information collected during the study will nonetheless be stored, analyzed, or used to protect the scientific integrity of the research project.

Any new findings that could influence your decision to stay in the research project will be shared with you as soon as possible.

ALTERNATIVE TREATMENTS

If you do not wish to take part in the study, your doctor will discuss your treatment options with you.

IDENTIFICATION OF CONTACT PEOPLE

If you have questions, or if you have a problem you think may be related to the study, or if you would like to withdraw, you may communicate with the Co-Investigator, Dr. Amal Baki during the day at (514) 890-8242 or with the research coordinator of the study at (514) 890-8000, extension 23224, 08:00 AM to 16:00PM, from Monday to Friday.

For any question concerning your rights as a research participant taking part in this study, or if you have comments, or wish to file a complaint, you may communicate with the local associate commissioner for complaints and service quality of the CHUM at 514-890-8484.

SIGNATURE

I have reviewed the information and consent form. Both the research study and the information and consent form were explained to me. My questions were answered, and I was given sufficient time to make a decision. After reflection, I consent to participate in this research study in accordance with the conditions stated above.

I authorize the research study team to have access to my medical record for the purposes of this study.

I authorize the researcher or his team to inform my treating doctor of my participation in this study and to communicate to him all relevant information.

YES 

NO 

Name (Please print) Signature of the participant to the research project Date

Name and contact information of the treating doctor:

Possibility to be contacted

| I am consenting to be contacted by the research team for a new research project which will be approved by the research ethics board. I will then be free to agree to participate or not. | ​​☐​ YES | ​​☐​ NO |
| --- | --- | --- |

SIGNATURE OF THE PERSON OBTAINING CONSENT, IF OTHER THAN THE INVESTIGATOR RESPONSIBLE FOR THE RESEARCH PROJECT

I have explained the research project ant the terms of this information and consent form to the research participant, and I answered all his/her questions.

Name (Please print) Signature of the participant obtaining consent Date

 COMMITMENT OF THE PRINCIPAL INVESTIGATOR AT CHUM

I certify that this information and consent form was explained to the research participant, and that the questions the participant had were answered.

I undertake, together with the research team, to respect what was agreed upon in the information and consent form, and to give a signed and dated copy of this form to the research participant.

Name (Please print) Signature of the principal investigator at CHUM Date

 WITNESS SIGNATURE

YES  NO 

Signature of the witness is required in the following cases:


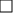
Difficulty reading or inability to read: The person (impartial witness) signing below attests that he/she has read the consent form and explained the project in detail to the participant, who appears to have understood it.


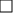
Not understanding the language, the consent form is in: The person signing below served as an interpreter for the participant during the consent process.

Name (Please print) Signature of witness Date

Please Note:

Any additional information about assistance given to the participant during the consent process must be noted in his/her research file.

APPROVAL OF THE RESEARCH ETHICS BOARD

The CHUM Research Ethics Board approved this study and is responsible for the monitoring for the institutions of the Québec Health and Social Services Network.
