# Supplementary material for: Evaluation of a Cannabis Harm Reduction Intervention for People With First-Episode Psychosis: Protocol for a Pilot Multicentric Randomized Trial
Source: JMIR Res Protoc. 2023 Dec 18;12:e53094. doi: 10.2196/53094 (PMC10758938; doi:10.2196/53094)
Supplement: Multimedia Appendix 2 [file resprot_v12i1e53094_app2.docx]

| **CHAMPS STUDY - Screening Form**  **Referral ID: ___ ___ ___**  **Participant Identifier:** ___ - ___ ___ - ___ ___ ___  **Visit Date:**  ___ ___ ___ ___ / ___ ___ / ___ ___ (yyyy/mm/dd)  **Visit #** ___ ___ |
| --- |

| **SECTION 1- Patient Referral Information (to be completed by clinic staff or referring psychiatrist)** | |  | | | | |
| --- | --- | --- | --- | --- | --- | --- |
| From which Clinic are you recruiting? | |  | | | | |
|  | JAP | |  | | |  |
|  | Connec~~-~~T | |  | | |  |
|  | NSEPP | |  | | |  |
|  | FMC-EPIP | |  | | |  |
|  | Clinique Notre Dame des Victoires | |  | | |  |
| Has the patient consented to be contacted for research purposes?  Yes  No | |  | | | | |
| If yes, what date was this consent obtained?  (DD/MMM/YYYY): __ __ / __ __ __ / __ __ __ __  What is the best way to contact the patient? | |  | | | | |
| \|  \| Phone call (able to leave message):  Phone number: _____________ \| \| --- \| --- \| \|  \| Phone call (DO NOT leave message) \| \|  \| Before clinic appointment \| \|  \| After clinic appointment \| \|  \| Email: ___________________________ \| \|  \| Other ____________________________ \|   Are there any special instructions for making contact with the patient (e.g., “don’t leave a message, stating where you are from”, “don’t use the patients name in text or emails”   \|  \| \| --- \| | |  | | | | |
| If the participant meets the inclusion and exclusion criteria and signs the consent form. He/she/they may be randomized to the Intervention group and will need a smartphone using one of the last 3 versions of iOS (version 10 and above) or Androids (version 8 and above). Can you confirm that the participant has the correct device, or we will need to lend him/her one for the duration of the study?  *Note: This will allow us to prepare for the randomization phase*. | |  | | | | |
| The participant has a smartphone with the adequate version to use the App. | |  | | | | |
| The participant doesn't have a smartphone. | |  | | | | |
| The participant has a smartphone but not the adequate version.  Unsure staff to confirm | |  | | | | |
|  | |  | | | | |
| **SECTION 2- Physician Screening** | |  | | | | |
| \| **INCLUSION CRITERIA** \| \| **Physicians confirm** \| **Staff confirm** \| \| --- \| --- \| --- \| --- \| \| 1. Is the patient a young adult aged between 18 and 35 years old? \| \| Yes  No \| Yes  No \| \| 2. Has the patient been diagnosed with any psychotic disorder? \| \| Yes  No \|  \| \| If yes, please specify which one here: \| \| \| \| \|  \| Schizophrenia \| \| \| \|  \| Schizoaffective disorder \| \| \| \|  \| Bipolar disorder with psychotic features \| \| \| \|  \| Brief psychotic disorder \| \| \| \|  \| Delusional disorder \| \| \| \|  \| Psychotic disorder not otherwise specified \| \| \| \|  \| Substance-induced psychotic disorder \| \| \| \|  \| Other \| \| \| \|  \| If [Other], Please Specify: _________ \| \| \| \|  \|  \| \| \| \| 3. Has this patient been followed at an early psychosis clinic for a minimum of 3 months? \| \| Yes  No \|  \| \| If Yes, please specify the date the patient started to attend the clinic (DD/MMM/YYYY): \| \| __ __ / __ __ __ / __ __ __ __ \| \| \| 4. Currently using cannabis (used cannabis at least once in the past month). \| \| Yes  No \| Yes  No \| \| 5. Open to changing cannabis-related practices. \| \| Yes  No \| Yes  No \| \| 6. Able to provide full informed consent \| \|  \| Yes  No \| \| 7. Available for the whole duration of the study/able to comply with study procedures \| \| Yes  No \| Yes  No \| \| 8. Able to comprehend French or English \| \| Yes  No \| Yes  No \| | |  | | | | |
| \| **EXCLUSION CRITERIA** \| **Physicians confirm** \| **Staff confirm** \| \| --- \| --- \| --- \| \| 1. Pregnancy or any medical condition that in the opinion of the psychiatrist precludes safe participation in the study or the ability to provide fully informed consent. \| Yes  No \| Yes  No \| \| 2. Any disabling, unstable or acute mental condition that in the opinion of the psychiatrist precludes safe participation in the study or the ability to provide fully informed consent. \| Yes  No \|  \| \| 3. Any legal/judicial status/issue, pending legal action, or other reasons in the opinion of the study team that might prevent completion of the study. \|  \| Yes  No \| \| 4. Presence of a substance use disorder that, in the opinion of the psychiatrist, precludes safe participation in the study (e.g., very unstable, or severe substance use disorder). \| Yes  No \|  \| \| 5. Currently seeking psychological or pharmacological treatment for CUD to cease or decrease his/her use. \| Yes  No \| Yes  No \| \| 6. Currently participating in another specific cannabis use-focused intervention. \| ☐ Yes ☐ No \| ☐ Yes ☐ No \| | | | | |  | |
| Report the total number of criteria checked in the DSM-5: | | | | |  | |
|  | 0-1: Does not meet criteria for Cannabis Use Disorder | | |  | | |
|  | 2-3: Mild Cannabis Use Disorder | | |  | | |
|  | 4-5: Moderate Cannabis Use Disorder | | |  | | |
|  | ≥ 6: Severe Cannabis Use Disorder | | |  | | |

**I have reviewed the Eligibility requirements for this participant in their entirety and acknowledge that this participant meets all medical inclusion and no medical exclusion criteria for the CHAMPS study.**

Signature of referring psychiatrist: ___________________ Date: ___________(yyyy/mm/dd)

**STAFF ELIGIBILITY CONFIRMED BY:**
