# Supplementary material for: Evaluation of a Cannabis Harm Reduction Intervention for People With First-Episode Psychosis: Protocol for a Pilot Multicentric Randomized Trial
Source: JMIR Res Protoc. 2023 Dec 18;12:e53094. doi: 10.2196/53094 (PMC10758938; doi:10.2196/53094)
Supplement: Multimedia Appendix 3 [file resprot_v12i1e53094_app3.docx]

***
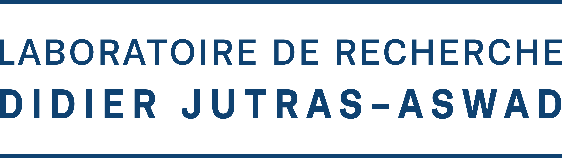

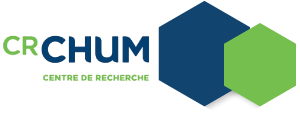
***

CONFIDENTIALITY POLICY

Translation from French version last update: April 2021

The CRCHUM and the research team of Dr. Didier Jutras-Aswad ("we") in collaboration with studio Akufen, attach great importance to the protection of your personal information and your privacy. We are committed to ensuring that your experience with us and the Application (the "Service") be as satisfactory and secure as possible.  You understand that by using the Service, it will be distributed to you by the CRCHUM and that any personal information you may provide to us may be used for the purposes set forth herein.

For the purposes of this Privacy Policy (the "Policy"), "Personal Information" means any information relating to an identified or identifiable individual. An identifiable individual is one who can be identified, directly or indirectly, by reference to one or more factors specific to his or her physical, physiological, genetic, mental, economic, cultural or social identity.

This Policy informs you of (i) the types of Personal Information that we process through the Service, (ii) the purposes for which it is processed and the legal basis for such processing, and (iii) how long we keep your Personal Information. This Policy also informs you of the categories of persons to whom we may transfer your Personal Information and under what circumstances we may do so.

In addition, you will find the rights you may exercise with respect to your Personal Information with us. We will treat your Personal Information only as set out in this Policy. We continually adjust our privacy practices to ensure that they comply at all times with the principles of the Personal Information Protection and Electronic Documents Act (Canada), the Act respecting the protection of personal information in the private sector (Quebec) and any other similar applicable legislation.

1. TYPE OF PERSONAL INFORMATION WE PROCESS

Personal Information we process when you create your account

When you create your account you must provide us with the following Personal Information: your name, email address and a telephone number.

Purposes and legal basis for processing

We process your name, email address, and phone number because you need to create your account. The legal basis for this processing is our legitimate interest in securing your account, the email is a unique identifier that will allow you to use the application, the phone number to contact you if you need assistance, and the first name will be used in the application for information purposes and by the caregiver to know who the person to be helped is.

We also process your email address to send you important messages about the Service, such as messages about the security of the Service and updates to this Policy. The legal basis for this processing is our legitimate interest in providing you with important security information or other important information about the Service or changes to this Policy. The legal basis for this processing is your consent. You may withdraw your consent at any time by contacting the Data Management Team also responsible for data protection, whose contact details for the Senior Data Manager appear below, or by clicking on the opt-out link at the bottom of our emails.

The Personal Information you provide to us through your use of the Service

The primary function of the Service is to provide a tool for you (whom we refer to as the "Recipient" for purposes of this Policy) to help change your cannabis use. At this time, the Service allows the research team (the "Researchers") to collect qualitative and quantitative data as well as automatically generated statistics as a result of using the Service. This data will be collected either to help/guide you to change your consumption or to answer our research questions.

Your case worker may be informed of your progress in using the service, but will not have access to qualitative data. You will be able to contact them if needed. We acknowledge that we are responsible, as clinical research personnel, for obtaining free and informed consent from the Beneficiary for the collection of quantitative and qualitative information about them.

Please note that you are not required to identify yourself, you are free to use a pseudonym or your study identifier (study number). However, it is the latter that will be used for informational purposes in the application and by the case worker. Your email address and a valid phone number are required, however, to access and use the service and to be contacted if you need assistance. Please note that this information is not transmitted to the researchers; in fact, the file of each Recipient is associated to a number and it is only to this identifier that the researchers have access, the data management team will be responsible for verifying the anonymity of the information.

At the end of each module, the mobile application will present you with content and resources to help you achieve your goals. In addition, a satisfaction questionnaire will be administered to you at the end of the intervention. The mobile application automatically generates a number of statistics related to the usage session (such qualitative, quantitative and statistical data are collectively referred to as "Data"). The Data so transmitted is stored on the Service's servers and will be accessible only by the data management team and used solely for research purposes.

Purposes and legal basis of processing

We process the Data that is submitted when you use the Service to make it accessible to Researchers so that it can be used for research purposes. The legal basis for this processing is the consent obtained from the Recipient.

We acknowledge our responsibility as clinical research staff to collect a free and enlightened consent from the Beneficiary to the collection of quantitative and qualitative information concerning them. If this consent should be withdrawn by the Beneficiary, we will have to stop using the Service in relation to this Beneficiary.

Purposes and legal basis of processing

If you have consented to the collection of information through cookies and other similar technologies, we will process these cookies and other similar technologies with the assistance of third party analytical services, for the purposes set forth in the section " COOKIES AND SIMILAR TECHNOLOGIES" section of this Policy. The legal basis for this processing is your consent. You may withdraw your consent at any time by contacting the data manager whose contact information is appears at the bottom of this Policy. Please be assured that that we do not associate any identifiers from cookies or similar technologies with information racial or ethnic origin, political opinions, religious or philosophical beliefs,  or your trade union membership, your health sexual orientation, or to your genetic or biometric data that could identify you.

Log files (Log)

We store log files on the Service's servers that include information such as your IP address, the date and time of your use of the Service, cookies that identify your browser and language, and various server requests and responses. These files may constitute Personal Information about you.

Purposes and legal basis for processing

We process log files relating to your use of the Service in order to understand the source of an error in the event of an error or bug in the Service and to establish statistics on connections to the Service. The legal basis for this processing is our legitimate interest in minimizing the number of interruptions and failures of the Service during your use of it.

2. CATEGORIES OF RECIPIENTS OF YOUR PERSONAL INFORMATION

Except as required by law or as expressly set forth in this Policy, we will never disclose your Personal Information to any third party.

Researchers and Case worker

When you use the Service, we provide your anonymized Data to Researchers, at the end of the study, or to perform preliminary analyses. Only your stakeholder will have access to your name, email and phone number throughout the study.

Service Providers

We will not provide any of your Personal Information to third parties who use it for their internal business functions (e.g., software development firms, customer service, maintenance, security, data analysis, emailing, beta testing or data hosting). In the event that certain third party service providers are asked to collect information, including Personal Information, on our behalf, these service providers will have agreed with us to comply with the laws applicable to your Personal Information and this Policy. We will only provide these service providers with the Personal Information they need to provide their services and they are prohibited from using this Personal Information for any other purpose.

Legal requirements

We may disclose your Personal Information if we have a good faith belief that such action is required by a subpoena, warrant, or other legal or administrative process served on us pursuant to law. We may also disclose Personal Information if we believe in good faith that such action is appropriate or necessary to prevent a breach of our terms or conditions of use, our user license agreements, or any other agreement to which you are a party; to protect against a claim to protect our rights, property, safety or those of a partner, person or the public; to maintain and ensure the security and integrity of the Service or our infrastructure from misuse or illegal use; to defend against claims or allegations by third parties; or to cooperate with governmental regulatory agencies having jurisdiction.

Transfer of ownership

Information about our users, including Personal Information, may be disclosed in connection with a bankruptcy, merger, sale or transfer of the business related to the Service, acquisition or similar transaction. In the event of such a transaction in which your Personal Information is to be transferred to a third party, we will use reasonable efforts to notify you. For example, we will post a notice of transfer of Personal Information on the Service and, if we have your email address, we will send a notice of transfer of Personal Information to that address. In addition, we will require the third party receiving your Personal Information in such a transfer to agree to protect the privacy of your Personal Information in a manner that is consistent with this Policy and to comply with applicable laws regarding Personal Information. They will also be required to agree to only process your Personal Information in accordance with this Policy unless they first notify you and, where required by law, obtain your consent.

Other cases

We may transfer your Personal Information to third parties if: (i) we have obtained your consent to do so, (ii) to our legal or other advisors, if they are subject to confidentiality obligations at least as stringent as those set out in this Policy and if they comply with applicable laws regarding Personal Information.

3. SECURITY OF YOUR PERSONAL INFORMATION

We take extensive measures, including the implementation and enforcement of physical, electronic and administrative procedures, to ensure the security, integrity and accuracy of all Personal Information collected. Our measures include procedures designed to prevent unauthorized access, modification, misuse or disclosure of Personal Information. In the event of a security breach, we may attempt to send you an email notification to allow you to take appropriate protective measures.

Notwithstanding the foregoing, data, including e-mail and Internet, network, telephone or other electronic communications may be unlawfully intercepted by unauthorized parties. We cannot guarantee the absolute efficiency and/or security of the Service. Among other things, it is possible for a hacker to gain access to the Service's servers by devious and illicit means. It is therefore important to keep in mind, before using the functionalities offered by the Service, that it is always possible that a malicious individual could access the Service's servers and use, for his or her own purposes, the information that you have disclosed on or through the Service.

4. RETENTION OF YOUR PERSONAL INFORMATION

We retain Personal Information only for as long as it is required to continue our research activities in a viable manner or as we deem legally required. Specifically, for as long as you maintain your account, we will retain the Personal Information requested from you for a period of five (5) years following your last login to the Service.

5. CONSENT

Before providing us with any Personal Information, you acknowledge that you have provided your consent to make such disclosure.

By providing us with Personal Information, you agree that we may collect or use such Personal Information in accordance with this Policy and the privacy preferences you have indicated to us, if any, and as permitted or required by law.

Subject to legal and contractual requirements, you may refuse or withdraw your consent to certain of the identified purposes at any time by contacting us. If you refuse or withdraw your consent, we may not be able to provide or continue to provide you with certain services or information that may be of value to you.

6. YOUR RIGHTS

Access to your Personal Information and portability

Generally, upon request, we will inform an individual of the existence, use and disclosure of his or her Personal Information and provide access to that information. An individual will be able to challenge the accuracy and completeness of the information and have it amended as appropriate.

Please note that access to your file containing your Personal Information is free of charge. However, we may charge you a reasonable fee for the physical reproduction and transmission of your Personal Information. If so, we will inform you of these costs in advance.

Without limiting the generality of the foregoing, you have the right to receive Personal Information about you that you have provided to us in a structured, commonly used and machine-readable format, you have the right to transmit such Personal Information to another data controller without our interference, where (i) the processing is based on your consent or on a contract between you and us and (ii) the processing is carried out using automated processes. In addition, where technically feasible, you may have such Personal Information transferred directly by us to your new data controller.

Accuracy of Your Personal Information and Right to be forgotten

The Personal Information you disclose to us shall be as complete, accurate and up-to-date as is necessary for the purposes for which it is to be used. You are entitled to have your file amended to correct inaccurate, incomplete or misleading information and to have outdated information or information that is not relevant to the purpose of the file deleted, or to provide written comments and have them placed on your file. Most of your personal information can be viewed and corrected by logging into your account and accessing your profile. Otherwise, we invite you to contact the Data Manager listed at the end of this Policy to request it.

Without limiting the generality of the foregoing, you have the right at any time to request, except in certain circumstances provided by law, the deletion of your Personal Information if: (i) it is no longer necessary for the purposes for which it was collected or otherwise processed; (ii) we have obtained your consent to process such Personal Information and there is no other legal basis for the processing; (iii) you object to the processing without us having a compelling legitimate reason for the processing, (iv) such Personal Information has been unlawfully processed; or (v) it must be erased to comply with a legal obligation under applicable law.

Limiting processing or the right to object to processing

You have the right at any time, in certain circumstances as provided by applicable law, to request from the Data Protection Manager whose contact details can be found at the end of this Policy that we limit certain processing we do of your Personal Information or to object to such processing.

Notices, complaints and communications

By publishing this Policy, we make readily available to individuals specific information about our policies and practices relating to the management of Personal Information. If you have any questions about this Policy, please feel free to contact the Data Manager, who is responsible for the Policy and can be reached at the address listed at the end of this Policy, so that we may respond to your questions to the extent possible. We reserve the right to ask you for certain information to identify you.

In the event of non-compliance with the principles set forth in this Policy, you may address a complaint to us by contacting the Data Manager. Depending on your jurisdiction (e.g. Canada and the European Union), you may also be able to file a complaint with a supervisory authority.

7. CHANGES TO THE POLICY

When we, in our sole discretion, make changes to this Policy, we will change the "Last Updated" date as found above.

While it is not our intention to make frequent or substantial changes to this Policy, we may do so to better serve you in the future or in light of changes in our service offerings, technology or the law. We encourage you to review this Policy regularly to be aware of any changes to the Policy. If the changes are material, we will send you a notice via the Service or other means so that you can review the changes before continuing to use the Service. If you do not agree with any of the changes, you must discontinue use of the Service. Your continued use of the Service following the posting or communication of such changes will constitute your acceptance of the revised Policy.

If you have any questions or comments regarding this Policy, please contact the Data Manager.
